# Developing and testing of an interactive internet-based intervention to reduce sexual harm of sexualised drug use (‘chemsex’) among men who have sex with men in Hong Kong: a study protocol for a randomised controlled trial

**DOI:** 10.1186/s12889-021-10742-8

**Published:** 2021-04-13

**Authors:** Edmond P. H. Choi, Pui Hing Chau, William C. W. Wong, Jojo Y. Y. Kowk, Kitty W. Y. Choi, Eric P. F. Chow

**Affiliations:** 1 School of Nursing, University of Hong Kong, 4/F, William M.W. Mong Block 21 Sassoon Road, Pokfulam, Hong Kong; 2 Department of Family Medicine and Primary Care, University of Hong Kong, Ap Lei Chau, Hong Kong; 3 Sticky Rice Love, Hong Kong, Hong Kong; 4 Melbourne Sexual Health Centre, Alfred Health, Melbourne, Victoria Australia; 5 Central Clinical School, Monash University, Melbourne, Victoria Australia; 6 Melbourne School of Population and Global Health, The University of Melbourne, Melbourne, Victoria Australia

**Keywords:** Chemsex, Men who have sex with men, Risky sexual behaviours, Sexual health, Sexualised drug use

## Abstract

**Background:**

Sexualised drug use, known as ‘chemsex’ or ‘chemfun,’ is the practice of intentionally using illicit drugs before or during sexual activates to enhance sexual arousal and pleasure. International and local data have both suggested that chemsex is common among men who have sex with men (MSM). Chemsex is generally seen with the engagement of risky sexual activities and therefore poses a threat regarding the potentially increased spread of human immunodeficiency virus and other sexually transmitted infections. However, little work has been done on the primary prevention of chemsex among MSM. Therefore, the aim of this study is to develop and evaluate an interactive internet-based intervention in reducing the sexual harms of chemsex among MSM in Hong Kong,

**Methods:**

A two-armed, randomised, parallel-group trial with a three-month follow-up period will be conducted. 250 MSM aged 18 years or the above will be recruited through local non-governmental organisations, social media and by snowballing in Hong Kong. Participants will be randomly allocated into either the intervention (*n* = 125) or control group (*n* = 125). The interactive internet-based intervention will be developed based on the theory of planned behaviours. Participants in the control group will receive a web-based intervention without any sexual health information and without any interactive components. The primary outcomes will be self-efficacy in refusing risky sexual behaviours and chemsex, as measured by the Drug Avoidance Self-Efficacy Scale, the Self-Efficacy for Sexual Safety and the Condom Self-Efficacy Scale. Subjects in both groups will be evaluated at baseline and 3 months after baseline.

**Discussion:**

To the best of our knowledge, this will be the first interactive internet-based intervention to specifically target chemsex among MSM. This project can help in the development and testing of culturally relevant health promotion programmes that reduce chemsex among MSM. Using an online delivery mode, the intervention is capable of reaching a large population of targets at a relatively low cost and thus has the potential to reduce the public health burden of chemsex and other risky sexual behaviours among MSM in a cost-effective manner.

**Trial registration:**

International standard randomized controlled trial number (ISRCTN) registry: ISRCTN20134522 registered on 17 March 2021.

## Background

### Chemsex: an emerging sexual health issue

Substance use is common among men who have sex with men (MSM). It is reported than MSM are three times more likely to use illicit substances than their heterosexual counterparts [1]. In Australia, about 60% of MSM used recreational drug in the past 6 months [2]. Another study in Melbourne, Australia found that 63.1% of MSM used at least one drug in the last 3 months and 36.6% used ≥2 drugs. Poppers (44.8%), marijuana (30.8%) and ecstasy (19.1%) were the three most common drugs used among MSM in the last 3 months [3]. Furthermore, the study found that 80.4% of ecstasy users and 74.3% of gamma-hydroxybutyrate (GHB) users reported the drug made them much more likely to kiss someone [3]. Another study in the United Kingdom found that one quarter of MSM had used three or more recreational drugs in the previous 3 months [4]. Recreational drug use is a global concern among MSM, particularly those who use drugs before or during sexual activities because it increases the likelihood of engaging in risky sexual behaviours [1].

Sexualised drug use, known as ‘chemsex’ or ‘chemfun,’ is the practice of intentionally using illicit drugs before or during sexual activates to enhance sexual arousal and pleasure. This practice, however, poses harmful risks to sexual health [1]. Chemsex is common among MSM [5]. One study in the Republic of Ireland found that 27% among 510 MSM reported engaging in chemsex within the previous 12 months [6], where a study in the Netherlands found that 41% of HIV-negative MSM and transgender persons who have sex with men (*n* = 330) had engaged in chemsex with casual partners in the previous 3 months [7].

Chemsex is also an emerging health issue in Hong Kong, where a report by the Hong Kong Jockey Club Drug Info Centre found that 17% of the surveyed MSM had ever engaged in chemsex, and more than half (58.18%) had engaged in their first chemsex experience between the ages of 16 and 25 [8]. A community-based organisation in Hong Kong also found that 9% of MSM respondents had ever engaged in chemsex [9], with those aged 31 to 35 being the most likely to engage in chemsex compared with other age groups. In Hong Kong, the most common drug used by MSM was methamphetamine (49%), followed by gamma-hydroxybutyrate (GHB)/ gamma-Butyrolactone (GBL) (27%), ecstasy (18%) and then ketamine (10%). Furthermore, 31% used methamphetamine and Viagra [9]. Additionally, an empirical study in Hong Kong found that that 8% of college students in Hong Kong had previously engaged in chemsex during their lifetime, and 73% of them were MSM [10]. Another study in Hong Kong reported that 12% of MSM had chemsex in the previous 6 months [11]. A qualitative study on 31 MSM in Hong Kong found that chemsex is also popular on dating apps [12]. The data suggest that there is a need to develop primary prevention programmes for chemsex among MSM.

### Dangers associated with chemsex

Severe drug interactions with a range of drugs, including human immunodeficiency virus (HIV) medications, can be fatal [13]. This is particularly relevant to MSM populations, which are disproportionately affected by HIV infections. Moreover, the use of pre-exposure prophylaxis to prevent the acquisition of HIV has become increasingly common among MSM [14]. In addition to drug interactions, the anaesthetic nature of some drugs can prolong the duration of sexual activities, which in turn can lead to rectal and penile trauma, potentially further facilitating the spread of sexually transmitted infections (STIs) [13]. Furthermore, because of the sexual disinhibition and hypersexuality induced by the drugs, chemsex has been found to increase the likelihood of engaging in other risky sexual behaviours, such as condomless anal sex, injecting drug use and higher alcohol consumption, fisting, sharing sex toys, and group sex [15]. This might increase the spread of HIV and other STIs [13, 16]. Indeed, one systematic review conducted in 2019 found the rates of condomless anal sex range from 30 to 38% among MSM who practiced chemsex [1]. Besides, a study in the United Kingdom reported that engaging in chemsex was associated with a higher number of condomless anal male partners [15]. A recent study in Australia also reported an association between condomless anal sex and chemsex among MSM with an odds ratio: 1.54 [17].

### Need of this trial

We propose this trial because chemsex, which is an emerging issue among MSM, substantially increases the risk of HIV and STIs infections. In spite of this, there have been very limited research evaluating the effectiveness of interventions or health promotion programmes which specifically aim to reduce the intention to engage in chemsex and the actual chemsex behaviours among MSM.

### Aim and objectives

This study aims to evaluate the effectiveness of the interactive internet-based intervention in reducing the sexual harms of chemsex among MSM, with the following four objectives:

1. To strengthen the self-efficacy in refusing risky sexual behaviours and chemsex.
2. To reduce the intention to engage in chemsex and the actual chemsex behaviours.
3. To enhance consistent condom use in both sober and drug-influenced sex.
4. To increase motivation towards the practice of regular HIV/STIs testing.

### Hypotheses

We hypothesise that, MSM receiving the internet-based intervention will be more likely to exhibit (i) better self-efficacy in refusing risky sexual behaviours and chemsex, (ii) lower intention to engage in chemsex and the actual chemsex behaviours, (iii) higher condom use consistency and (vi) more HIV and STI testing, compared to MSM in the control group.

## Methods

### Study design

This is a two-armed, randomised, parallel-group trial with a three-month follow-up period.

### Participants

The study inclusion criteria are (i) MSM, (ii) cis male, (iii) adult (aged 18 years or older), and (iv) having internet access. People who cannot read and understand Chinese will be not be eligible to the study.

### Sample size justification

The sample size is determined based on G*Power, using an independent t-test, to detect a small-to-moderate between-group difference (Cohen’s d = 0.4) in the primary outcome. A minimum sample size of 200 MSM (100 in each group) will be needed to achieve a power of 80% at a 0.05 level of two-sided significance. We anticipate that 20% of the participants will drop out, we will recruit additional 50 (25 in each group) MSM. Therefore, a total of 250 MSM will be recruited.

### Sampling frame

To ensure that we can recruit the required number of study participants with a diverse background, we will use multiple recruitment sources for participant recruitment. First, we will collaborate with local non-governmental organisations that target MSM populations in Hong Kong. They will recruit potential participants based on their existing network. Second, we will recruit participants by using social media (such as Instagram and Twitter) and other online platforms (such as forums) that target MSM. Third, we will use snowball sampling for recruitment. In other words, the enrolled participants will be asked to invite and refer potentially interested friends to participate the study. We have used these recruitment strategies to recruit MSM in previous studies, confirming the feasibility of these recruitment methods [18]. It is anticipated that it will require 6 months to recruit 250 MSM.

### Online enrolment and consent

We will develop a website for study enrolment. A screening questionnaire will be used to determinate whether the potential participants meet the criteria to participate in the study. Eligible participants will be requested to sign an electronic consent form confirming the study details. They also need to provide contact information. After completing these steps, participants will be asked complete an online baseline questionnaire.

### Randomisation and allocation concealment

After completing the baseline questionnaire, participants will be randomly assigned to either the intervention group or control group via a computer-generated block randomisation procedure (with a block size of four for two groups) with a 1:1 randomisation ratio. The computer-generated sequence will be generated by an independent researcher who is not involved in the trial. There will be no stratification. All participants and research team members are blind to allocation sequence. After randomisation, study participants will be automatically guided to the web content associated with their allocation. The participants will be blind.

### Intervention group

A meta-analysis of HIV behavioural interventions aimed at reducing the sexual risk behaviour of MSM concluded that interventions using a theoretical basis such as the diffusion of innovations theory and the model of relapse prevention were associated with significantly greater reductions in condomless anal sex than interventions not reporting a theory with an odds ratio: 0.65 [19]. Thus, it is important to develop interventions and health promoting programmes based on theoretical frameworks.

The theory of planned behaviours (TPB) will be used in the current trial to guide the intervention development. According to the TPB, behaviours are primarily determined by intention and perceived control. It theorises that attitudes, perceived norms and behavioural control predict intention [20]. We will employ the TPB in our trial based on the evidence from past studies. First, one meta-analysis reported that the TPB is a strong and appropriate model for predicting sexual behaviours among MSM [21]. Another meta-analysis also supported the use of the TPB as a theoretical framework for designing interventions to reduce sexual risks [22]. Furthermore, a previous study in mainland China found that the TPB could be used to predict syringe sharing behaviours among female injecting-drug users who were also sex workers [23]. A more recent study among MSM in Hong Kong report that three constructs of the TPB measured at baseline were significantly associated with chemsex during the 6-month follow-up period. These constructs included: (i) positive attitudes toward chemsex (adjusted odds ratio: 1.19), (ii) perceived support for chemsex from significant others (adjusted odds ratio: 1.15), and (iii) perceived behavioural control of refraining from chemsex (adjusted odds ratio: 0.76) [24]. Therefore, the TPB is recommended for the present trial.

Rather than adopting a traditional anti-drug approach to combat drug use with sex, this project proposes to intervene from the perspective of harm reduction, primarily with regards to sexual harm. In fact, people engage in chemsex for multiple reasons. While it is difficult for current chemsex users to withdraw drug use in a short period, being empowered to carry out safer sex practices even in drug-influenced situations may be a more effective and realistic way to lower the risks regarding HIV/STI transmission from chemsex. Self-efficacy of such can be further extended to contexts of sober sex, which would also benefit sexual health at the community level. Based on the TPB, the intervention’s aims are:

- To lower the desire to use drugs at sex by enhancing participants’ knowledge of chemsex, including negative health impacts, risks, social stigmas and legal consequences;
- To foster a positive attitude towards consistent condom use;
- To foster a positive attitude toward regular HIV/STI testing;
- To increase the self-efficacy of refusing chemsex;
- To improve participants’ perceived self-efficacy in making informed decisions on HIV/STI prevention in both sober and drug-influenced sex; and
- To set expectations that consistent condom use and regular HIV/STI testing are normative.

We propose an interactive internet-based intervention for the following rationales. First, a Cochrane Review found that interactive computer-based interventions (ICBIs) are effective for sexual health promotion, while also having positive impacts on self-efficacy, intention and sexual behaviours [25]. The definition of ‘interactive intervention’ is that interventions comprise components that require user contributions (e.g. completing knowledge tests, entering personal data and making choices) to produce tailored feedback that is personally relevant to users [26]. Furthermore, the review also found that compared with face-to-face sexual health interventions, ICBIs are more effective [25]. Second, another systematic review and meta-analysis also reported that computer technology-based HIV prevention interventions are effective in reducing risky sexual behaviours [27]. The review also recommended computer technology-based HIV prevention interventions because of their low cost to deliver, the customisability of intervention content and the flexibility of dissemination channels [27]. Third, a systematic review also showed evidence that eHealth for HIV prevention in high-risk MSM has the potential to be effective in the short term for reducing HIV risk behaviours and increasing testing rates [28]. Fourth, a systematic review of mobile-based interventions (including web-based and smartphone application-based interventions) concluded that mobile-based technology is a promising method for addressing substance abuse because the interventions have been shown to reduce substance use among vulnerable individuals [29]. These four systematic reviews provide compelling evidence to support the use of internet-based interventions to prevent chemsex among MSM.

Internet-based interventions have several strengths. First, sexual health, homosexuality and chemsex are considered embarrassing topics in many cultures including the Chinese culture. Internet-based interventions can provide greater privacy and anonymity than traditional face-to-face interventions. Second, unlike traditional face-to-face interventions, participants can access internet-based interventions at their convenience. Third, web-based platforms facilitate data collection. Fourth, the dissemination of web-based interventions is fast and relatively cheap. Finally, and more importantly, in terms of empirical evidence, multiple systematic reviews support the effectiveness of eHealth interventions in promoting HIV-preventive behaviours among MSM [30–32]. Therefore, a web-based intervention is recommended for the proposed study.

The time spent on the web-based intervention is expected to be approximately 20 to 30 min for the intervention group. Reminders (via email or instant messaging such as WhatsApp) will be sent to the participants to ask them to access to the web-based intervention again at month 2 to enhance the intervention effect.

### Control group

Participants in the control group will receive a web-based intervention without any sexual health information and without any interactive components. The contents for the control group will only include educational materials on general health information. Reminders (via email or instant messaging such as WhatsApp) will be sent to the participants to ask them to access to the web-based intervention again at month 2.

### Access to the allocated web content

The study participants will have unlimited access to their allocated contents during the study period. However, the contents will only be available to participants with a registered email address and password to minimise contamination between intervention and control participants. In other words, if the participants need to gain access to their allocated web contents during the study period, they will need to use their registered email address and password to log in.

### Study outcomes

The study outcomes in both groups will be measured at baseline (T0) and at 3 months (T1) after baseline. The primary outcomes will be self-efficacy in refusing risky sexual behaviours and chemsex. The secondary outcomes will be (i) intention to have chemsex, (ii) actual chemsex behaviours, (iii) condom use consistency in both sober and drug-influenced sex and (iv) having regular HIV/STI testing.

### Study instruments

As there are no existing study instruments to specifically measure self-efficacy in refusing chemsex and risky sexual behaviours, we use (i) the Chinese version of Drug Avoidance Self-Efficacy Scale (DASES), (ii) the Self-Efficacy for Sexual Safety scale and (iii) the traditional Chinese version of the Condom Self-Efficacy Scale (CSES).

- DASES was developed by Martin et al. [33] to measure drug avoidance self-efficacy. There are 16 items assessing abstinence self-efficacy across different high-risk situations. For each question, study participants are asked to imagine themselves in a particular situation and rate their perceived level of self-efficacy in resisting drug use in that situation. One example of the questions asked is: “Imagine that you are going to a party where you will meet new people. You feel that drug use will relax you and make you more confident. Could you avoid drug use?” A seven-point Likert scale (1 = certainly no to 7 = certainly yes) is used. The total score is obtained by summing across the 16 items, with a higher total score indicating a high level of self-efficacy to resist drug use [33].
- Self-Efficacy for Sexual Safety consists of seven items. Participants are asked to rate on a five-point scale ranging from ‘strongly disagree’ to ‘strongly agree’ regarding a number of statements. Examples of the statements include: “When I am drunk or high, I can avoid situations that I consider sexually risky” and “When I am drunk or high, I can choose safer sex with a man I have never had sex with before” [34]. A higher score indicates a higher level of self-efficacy for safe sex.
- CSES has 14 items, covering three subscales: (i) consistent condom use, (ii) correct condom use and (iii) communication. Participants will be asked to rate themselves on a five-point scale ranging from ‘very unsure’ to ‘very sure’ [35]. A higher score indicates a higher level of self-efficacy for condom use.

For the secondary outcomes, participants’ (i) intention to have chemsex, (ii) actual engagement in chemsex, (iii) practice of condomless sex both sober and drug-influenced, and (iv) the number of HIV/STI tests in the previous 3 months will be assessed.

Sociodemographic factors such as age and education level will be collected. Participants will be asked about their relationships and sexual behaviours to investigate elements such as preferred sexual position (top/bottom/versatile), frequency of condom use, number of sexual partners, relationship status, etc.

Table 1 shows the SPIRIT diagram for the schedule of enrolment, intervention and assessment.

**Table 1:** SPIRIT diagram for the schedule of enrolment, interventions, and assessments

	Study Period			
	Enrolment	Allocation	Post-allocation	Close-out
Timepoint^b^	***-t***_***1***_	0	***T0***	***T1***
**Enrolment:**				
**Eligibility screen**	X			
**Informed consent**	X			
**Allocation**		X		
**Interventions:**				
***Intervention group***			X	
***Control group***			X	
**Assessments:**				
***Primary outcome and secondary outcomes***^a^	X			
***Primary outcome and secondary outcomes***^a^				X

^a^The primary outcomes will be self-efficacy in refusing risky sexual behaviours and chemsex. The secondary outcomes will be (i) intention to have chemsex, (ii) actual chemsex behaviours, (iii) condom use consistency in both sober and drug-influenced sex and (iv) having regular HIV/STI testing

^b^The study outcomes will be measured in both groups at baseline (T0) and at 3 months (T1) after baseline

### Data analysis

All continuous outcomes will be analysed using the linear mixed-effects model, with the intervention group as the independent variable and adjusting for baseline characteristics as covariates. Similarly, a generalized linear mixed-effects model with logit link will be used to analyse the binary outcomes. The intention-to-treat principle will be adopted, and all study subjects will be randomised and included in the analysis. Missing values at the three-month follow-up will be handled by the model without the need of imputation.

## Discussion

International and local data have both suggested that chemsex is not uncommon among MSM. The negative impacts of chemsex is multifaceted including mental health, increased risks of HIV and STIs, and social functioning [1]. Importantly, chemsex is in general carried out alongside other risky sexual activities and therefore poses a threat regarding the spread of HIV and other STIs in the community [1]. However, compared with other areas, such as promoting condom use among MSM and drug prevention among the general population, little work has been done on the primary prevention of chemsex among MSM, particularly in Hong Kong.

To the best of our knowledge, this will be the first interactive internet-based intervention specifically targeting chemsex among MSM. This project can help to develop and test culturally relevant health promotion programmes in reducing chemsex among MSM. This internet-based intervention, if found successful, will have important clinical and policy implications, as it could be adopted by governmental and non-governmental organisations targeting MSM. The programme will also raise the awareness of MSM regarding safe sex practices such as the consistent use of condoms and the importance of having regular HIV/STI check-ups. Eventually, they may be empowered to reduce their participation in risky sexual behaviours, leading to the decrease in HIV/STI transmission in the MSM community.

Using an online delivery mode, the intervention is capable of reaching a large population of targets at a relatively low cost. As such, it has the potential to reduce the public health burden of chemsex and other risky sexual behaviours among MSM in a cost-effective manner. Ultimately, we expect this intervention could be modified and implemented throughout the entire Hong Kong community.

### Limitation

First, the study will be conducted in the Chinese population only. The applicability of the study findings in other populations is uncertain. Second, the follow-up period is relatively short. The long-term effectiveness of the intervention is therefore unknown. The acceptability of this internet-based intervention among people with low computer literacy is not sure.

## Conclusion

This programme is the first attempt to develop an online intervention to reduce sexual harm related to chemsex among MSM. The study will provide evidence that is readily translatable to real-life practice through a territory-wide campaign promoting sexual health. If the online intervention is effective, it could be adopted as a relatively low-cost, sustainable community-based health promotion strategy by sexual health clinics, as well as community centres that target MSM. We expect the study to have a clinical impact on future development and implementation of online interventions for MSM both locally and globally.

## Data Availability

The data sharing plans for the current study are unknown and will be made available at a later date.

## Abbreviations

CSES: Condom Self-Efficacy Scale
DASES: Drug Avoidance Self-Efficacy Scale
HIV: Human immunodeficiency virus
ICBIs: Interactive computer-based interventions
ISRCTN: International standard randomized controlled trial number
MSM: Men who have sex with men
RCT: Randomised control trial
STIs: Sexually transmitted infections
TPB: Theory of planned behaviour
